# Is Combination Therapy for Chronic Hepatitis C Toxic for Cardiac Function?

**DOI:** 10.5812/hepatmon.6254

**Published:** 2012-08-20

**Authors:** Ramzy Almawardy, Walid Elhammady, Nasser Mousa, Sherif Abotaleb

**Affiliations:** 1 Cardiology Department, Ain Shams University, Egypt; 2 Tropical Department, Mansoura University, Mansoura, Egypt

**Keywords:** Hepatitis C, Chronic, Cardiovascular System, Echocardiography

## Abstract

**Background:**

Many types of cardiovascular complications such as; cardiac arrhythmias, impaired cardiac function, myocardial ischemia and decreased left ventricular function, have been attributed to interferon therapy.

**Objectives:**

The aim of this study was to evaluate the effects of combination therapy pegylated interferon and ribavirin on left ventricular systolic and diastolic functions in patients with a chronic hepatitis C infection.

**Patients and Methods:**

A total of 120 patients, eligible for hepatitis C virus (HCV) treatment with pegylated interferon and ribavirin, were included in this study. All patients underwent a full cardiovascular baseline examination including; detailed medical history, thorough clinical examination, 12 lead electrocardiogram (ECG), and echocardiography. A cardiac evaluation was performed at the beginning and six months after starting combination therapy.

**Results:**

No significant changes regarding cardiac symptoms including; shortness of breath, cough, palpitations, chest pain and hypertension, were found during or six months after starting the combined therapy. ECG findings showed statistically non-significant decreases in the QT interval, while corrected QT showed statistically non-significant increases six months after beginning combined therapy, when compared to their values before treatment. Also with regard to the echocardiography findings, there was no statistically significant difference found between any of the echocardiography parameters six months after starting combined therapy compared to their values before treatment.

**Conclusions:**

The results of our study suggest that, combination therapy does not cause a significant deterioration in cardiac function in patients with a chronic hepatitis C infection, and it may be used safely in patients without cardiac disease.

## 1. Background

It is estimated that approximately 130–210 million individuals, i.e., 3% of the world’s population, are chronically infected with the hepatitis C virus (HCV) (1, 2). Chronic HCV infections are endemic in Egypt, and based on the results of Ray et al., approximately 91% of Egyptian patients with chronic HCV, are infected with HCV genotype 4 (3). The first line of treatment for chronic hepatitis C is based on the use of one of the two pegylated interferon-alpha formulations, administered subcutaneously weekly, in combination with daily oral ribavirin (4, 5, 6). Adverse side effects of interferon include; a flu-like syndrome, myelosuppression, gastrointestinal problems (anorexia, nausea, vomiting, diarrhea, and transaminase elevation), neurological (somnolence and confusion) and dermatological toxicity. Interferon has also been known to induce adverse cardiac effects (7). Cardiovascular effects related to interferon-alpha have been reported in 5 to 15% of patients (8). The most common cardio-toxic clinical effects of interferon are; arrhythmias (58%), acute coronary syndrome (21%), cardiomyopathies (12%) and other manifestations, including pericarditis (9%) (9). It has also been shown that human recombinant interferon alfa induces conduction slowing and ventricular arrhythmias (10).

## 2. Objectives

The aim of this work was to investigate the effects of combination therapy, pegylated interferon and ribavirin, on the left ventricular systolic and diastolic functions in patients with chronic hepatitis C.

## 3. Patients and Methods

### 3.1. Patients

This study was carried out on 165 Egyptian patients who had a chronic HCV infection. The patients attended the Damietta Cardiology and Gastroenterology Center, and they were candidates for treatment with combination therapy pegylated interferon and ribavirin, between April 2011 and March 2012. The dosage of pegylated interferon alfa-2a was 180 mcg injected subcutaneously once weekly, and the pegylated interferon alfa-2b dosage was 1.5 mcg/Kg. All patients were treated in addition to the pegylated IFN, with ribavirin; this was administered in orally divided doses at 1000 mg⁄ day and 1200 mg⁄ day in patients weighing < 75 kg and > 75 kg respectively. Patients were educated on the potential complications, especially cardiac symptoms and were assessed for safety, tolerance and efficacy of the therapy, this occurred weekly for the first month and then every month till the end of their treatment. Cardiac examinations were carried out before the start of treatment and six months after initiation of treatment. Exclusion criteria included; decompensated liver cirrhosis (ascites, encephalopathy, bleeding varices), patients with F0 and F4 on the METAVIR scoring system (they were not included in our Egyptian protocol for treatment of chronic hepatitis C), auto-immune hepatitis, chronic hepatitis B, combined chronic hepatitis B and C, patients with uncontrolled psychiatric disorders, cardiac disease (cardiomyopathy, arrhythmias, ischemia, myocarditis, and significant valvular disease), advanced renal impairment, uncontrolled thyroid dysfunction, poorly echogenic patients and pregnancy. Also patients with diabetes mellitus and hypertension were excluded. In total, 45 patients were excluded, 15 patients neglected their cardiac follow-up, and 30 patients were non-responders (patients who failed to achieve a decline of 2 log HCV RNA IU/ml after 12 weeks of treatment or who never achieved undetectable HCV RNA during treatment of a minimum duration of 24 weeks) and refused to continue the study. The study protocol conformed to the ethical guidelines of the 1975 Declaration of Helsinki. Informed consent was obtained from all patients. All patients included in the study were subjected to the following.

#### 3.1.1. History Taking

History included; name, age, sex, occupation, and residence, shortness of breath, cough, hemoptysis, palpitations, chest pain, hypertension, diabetes mellitus and paroxysmal nocturnal dyspnea.

#### 3.1.2. Clinical Examination

Measurements were taken of the patient’s; blood pressure, pulse, jugular venous pressure, pallor, jaundice, cyanosis, ascites, edema of the lower limbs, S3, S4 and any detected murmurs.

### 3.2. Investigations

#### 3.2.1. Blood Biochemistry and Cellular Parameters

Liver function tests included; aspartate aminotransferase (AST), alanine aminotransferase (ALT), serum bilirubin, serum albumin, alkaline phosphatase, prothrombin time and INR.

Blood tests included; serum creatinine; blood glucose (fasting and postprandial blood sugar), complete blood count (hemoglobin, white blood cells, red blood cells and platelets).

#### 3.2.2. Enzyme-Linked Immunosorbent Assay (ELISA)

Viral markers for hepatitis A virus (HAV), hepatitis B virus (HBV), HCV and HIV, were screened using an ELISA technique, (ELISA Kit, Abbott Diagnostics). Thyroid stimulating hormone (TSH), fT3, T4 and alpha fetoprotein (AFP) were studied with the ELISA method using Abbott laboratory reagents, USA (normal level of AFP was defined as < 8.1 ng/mL), autoantibodies; anti-nuclear antibody.

#### 3.2.3. Histopathology of Percutaneous Liver Biopsy

A percutaneous liver biopsy (≥ 15 mm in length) was performed on all of the patients. Liver biopsies were paraffin-embedded and stained with hematoxylin, eosin and Masson’s trichrome stains; additional stains were used when required. The biopsies were reviewed by a single pathologist. Hepatitis grading and staging were evaluated according to the Metavir scoring system, grading of the Metavir system is simply classified as (A0 to A3) and the stage of liver fibrosis (F0-F4) (11).

#### 3.2.4. Abdominal Ultrasound

An abdominal ultrasound was used to assess for liver cirrhosis, splenomegaly, or ascites, and to check the kidneys and pancreas.

#### 3.2.5. Cardiac Examination

ECG; a standard 12 lead ECG was recorded pre-treatment and six months after starting the treatment to document the presence of significant ST changes suggestive of ischemic heart disease, with assessment of the QT interval and corrected QT interval.

Echocardiography; a full 2-D, M-mode, Doppler and color flow mapping echocardiography study (Vivid 3 machine, GE3.5 probe) was performed pre-treatment and six months from the start of the treatment course. The following were estimated; end diastolic dimension (EDD), end systolic dimension (ESD), interventricular septum thickness (IVS), posterior wall thickness (PWT), left atrial diameter, and mitral regurgitation (MR). Systolic function was assessed by; ejection fraction (EF) % by M-mode in parasternal short axis view and 2-D mode in apical four chamber view by the Simpson’s method, fractional shortening (FS) % diastolic function was assessed by; E/A ratio, isovolumetric relaxation time, and deceleration time.

### 3.3. Statistical Analysis of Data

The collected data were organized, tabulated and statistically analyzed using SPSS software computer package (version 16). For the qualitative data; frequency and distribution percentage were calculated and for comparison between values before and after treatment the Wilcoxon test was used. For the quantitative data; mean, SD, minimum and maximum were calculated and for comparison between values before and after treatment, a paired sample t-test was used. P value < 0.05 was considered to be significant.

## 4. Results

**Figure 1:**
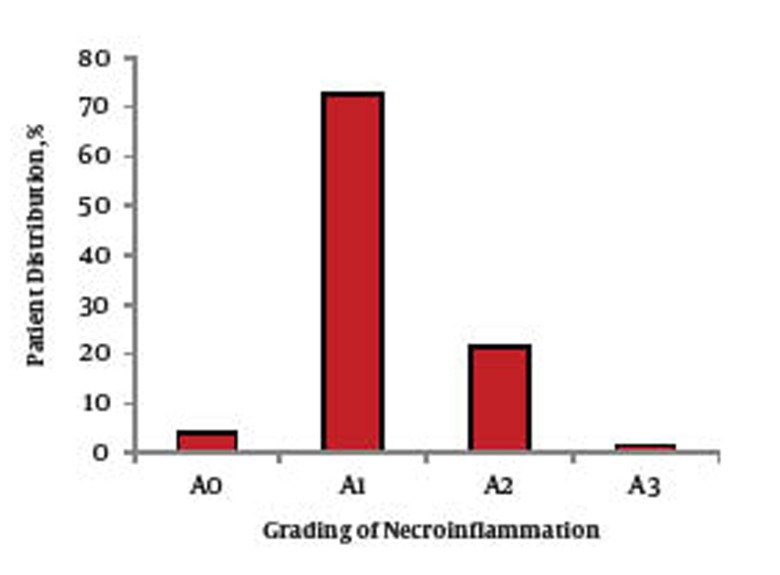
Distribution Percent of Studied Cases According to Grading of Necriinflammation

**Figure 2:**
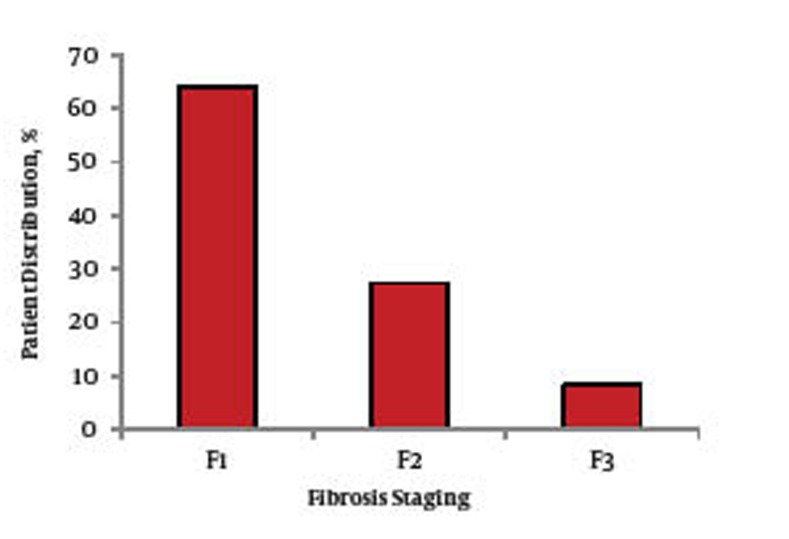
Distribution Percent of Studied Cases According to Fibrosis Staging

The 120 patients who fulfilled the inclusion criteria, continued to participate in this study. A total of 58 patients (48.33%) were given pegylated interferon alfa-2a, while the other 62 patients (51.66%) took pegylated interferon alfa-2b. The studied group consisted of 64 males (53.3%) and 56 females (46.7%) with a mean age of (43.4 ± 10.9). In the present study, their ages ranged from 23 to 59 years with a mean of 44.98 ± 8.55 years and the difference between males and females was statistically insignificant (45.02 ± 8.57 in females compared to 44.96 ± 96 in females). The biochemical characteristics of all the patients included in the study are shown in Table 1. Out of the 120 patients who continued in our study, no cases showed a reduction in their hemoglobin level which required cessation of ribavirin medication (if hemoglobin level is less than 8.5 g/dl, a reduction of the ribavirin dose by 200-400 mg/day is required), or discontinuation of both components of combination therapy (if hemoglobin level is less than 7 g/dl). In regard to the hepatitis grading and staging according to the Metavir scoring system, Figure 1 shows A0 in five patients (4.15%), A1 in 87 patients (72.5%), A2 in 26 patients (21.6%) and A3 in two patients (1.6%). Figure 2 shows F1 in 77 patients (63.9%), F2 in 33 patients (27.39%), F3 in 10 patients (8.30%) and no patients included in our study had F4. There were statistically insignificant increases in; shortness of breath, cough, palpitation chest pain and hypertension six months after starting combination therapy compared to those before treatment (Table 2). Regarding the ECG findings, the QT showed statistically non-significant decreases, while corrected QT showed statistically non-significant increases six months after starting combination therapy, when compared to their values before treatment (Table 3). There was no statistically significant difference between any of the echocardiography parameters six months after starting combination therapy compared to their values before treatment (Table 4).

**Table 1:** Laboratory Findings among the Study Group

	**Mean ± SD**
SGOT, U/L	52.07 ± 26.98
SGPT, U/L	47.30 ± 22.96
Bilirubin, mg/dl	0.88 ± 0.48
Albumin, g/L	4.26 ± 0.33
Creatinine, mg/dl	0.85 ± 0.16
Fasting BS, mg/dl	109.1 ± 34.58
Alpha fetoprotein, U/L	5.37 ± 6.49
Hemoglobin, g/dl	14.64 ± 1.26
WBCs (× 10^9^/L)	6.01 ± 1.87
Platelets (× 10^9^/L)	189.62 ± 55.18

Abbreviations: BS, blood sugar; SGOT, serum glutamic oxaloacetic transaminase; SGPT, serum glutamic pyruvic transaminase; WBCs, white blood cells

**Table 2:** Incidence of Symptoms in Patients Before and Six Months After Starting Combined Therapy

	**Pre-Treatment, No. (%)**	**Six Months After Starting Combination Therapy, No. (%)**	**P value**
Shortness of breath	0 (0)	3 (2.5)	0.083
Cough	0 (0)	2 (1.7)	0.15
Palpitation	0 (0)	3 (2.5)	0.083
Chest pain	0 (0)	2 (1.7)	0.15
Hypertension	0 (0)	3 (2.5)	0.83

**Table 3:** Comparison of QT and QTc in Patients Before and Six Months After Starting Combined Therapy

**Variable **	**Pre-Treatment, Mean ± SD**	**Six Months After Starting Combination Therapy, Mean ± SD**	**P value **
QT	362.92 ± 32.42	360.17 ± 36.80	0.15
QTc	399.52 ± 23.41	402.40 ± 22.56	0.06

**Table 4:** Changes in Echocardiographic Findings in Patients before and Six Months after Starting Combined Therapy

	**Pre-treatment, Mean ± SD**	**Six Months After Starting Combination Therapy, Mean ± SD**	**P value **
EDD, cm	4.89 ± 0.51	4.80 ± 0.63	0.15
ESD, cm	3.25 ± 1.96	2.98 ± 0.42	0.14
IVS, mm	9.52 ± 1.54	9.53 ± 1.49	0.94
PWT, mm	9.22 ± 1.81	9.19 ± 1.62	0.85
LAD, mm	32.68 ± 3.59	33.05 ± 3.23	0.23
LVEF, %	71.14 ± 5.38	71.41 ± 5.95	0.63
FS, %	37.90 ± 5.89	38.13 ± 5.92	0.61
E/A ratio	1.17 ± 0.30	1.18 ± 0.26	0.27
IVRT, msc	75.90 ± 16.09	73.50 ± 14.74	0.9
Deceleration time, msc	259.89 ± 82.37	254.72 ± 84.54	0.43

Abbreviations: EDD, end diastolic dimension; ESD, end systolic dimension; FS, fractional shortening; IVRT, isovolumetric relaxation time; IVS, interventricular septal thickness; LAD, left atrial dimension; LVEF, left ventricular ejection fraction; PWT, left ventricular posterior wall thickness

## 5. Discussion

The HCV epidemic has been particularly devastating in Egypt, where the prevalence of HCV infection is estimated to be 12% of the total population (12). Myocardial impairments for which a causal relationship with HCV infection has been suspected to date includes; dilated cardiomyopathy, hypertrophic cardiomyopathy, and arrhythmogenic right ventricular dysplasia cardiomyopathy, and chronic myocarditis (13). Pegylated interferon (Peg-IFN) in combination with ribavirin therapy is currently the first-line treatment option for chronic hepatitis C. This regimen can achieve sustained virological response, in more than 50% of chronic hepatitis C patients (14). On the other hand, interferon is also known to induce adverse cardiac effects such as ventricular LPs in patients with chronic active hepatitis (15). Interferon can also cause; flu-like symptoms, hypotension or hypertension, and tachycardia after treatment (16). Risk factors for interferon-induced cardio toxicity are not clearly understood and are not associated with age or dose because toxicity can occur at both low and high daily doses. Patients with a previous history of coronary artery disease may be at risk of ischemic changes, because the interferon flu-like reaction accompanied with fever causes an increased myocardial oxygen demand, but the exact mechanism is not clearly understood. So only a prior history of coronary artery disease has been identified as a possible risk factor to interferon-induced arrhythmia and ischemia (17). As regards to the ECG findings, the QT showed statistically non-significant decreases after treatment when compared to their values before treatment, while corrected QT values showed statistically non-significant increases after treatment when compared to their values before treatment. Previous studies have demonstrated that, no significant changes in heart rate and in frequency of ventricular or supraventricular ectopic beats occurred when comparing monitoring at baseline and during therapy. It seems therefore, that even arrhythmia is a very uncommon side effect of interferon therapy (18).

The echocardiography findings showed no statistically significant differences between any of echocardiography parameters following treatment compared to their values before treatment. In agreement with results of the present study, Erol et al. reported that there was a statistically non-significant increase in E/A ratio from 1.36 ± 0.34 before interferon therapy to 1.40 ± 0.50 after interferon treatment. They also reported that, there was no difference after treatment as regard deceleration time and EF in comparison to their values before treatment (19). Our study demonstrated no statistically significant difference regarding the left ventricular end-diastolic dimension (LVEDD) and left ventricular end-systolic dimension (LVESD) after interferon therapy. In disagreement with these results, Pauschinger et al. reported that there was a statistically significant decrease in LVEDD and LVESD after interferon therapy in comparison to their values before treatment. They also reported statistically significant increases in EF after interferon treatment in comparison to their values before treatment. A possible explanation for this contradiction may be attributed to the fact that, Pauschinger et al. investigated changes in viral myocarditis, regardless of the viral cause which can be seen from the increased values of LVESD and LVEDD present from the beginning. They attributed these beneficial effects to the reduction of viral load and effects on the myocardium (20). There are also some other case reports of interferon-induced cardiomyopathy (7). Our study indicated that IFN therapy did not induce any significant changes in left ventricular systolic or diastolic function. In agreement with our results, Kadayifci et al. reported that no significant changes or adverse effects were detected in the clinical examination, or in the electrocardiographic and echocardiographic evaluations during and after IFN therapy in patients with hepatitis (21). However, Sartory et al. (22) reported that a significant left ventricular ejection fraction decreased when viewed by radionuclide angiography, this appeared after one month of interferon therapy, but the effect disappeared after cessation of the therapy. The cause of the difference between our results and the results of Sartory et al. is not clear, however, it might be explained by differences in the patients’ characteristics, the number of patients, no follow up after one month, and different techniques (echo versus radionuclide angiography). In our study no case required the cessation of combined therapy due to cardiovascular complications. A very similar result was obtained by Kouno et al. who reported that, cardiovascular complications requiring the cessation of interferon administration were observed in only 0.62% of 643 treated chronic hepatitis C patients (23). Since we did not use cardiac troponin and myocardial perfusion scintigraphy, which are more sensitive for the detection of myocardial injury, the results of our study cannot fully exclude myocardial involvement, but our findings suggest that this type of therapy does not seem to affect cardiac function.

This study has some limitations which include; the small sample size of cases, incomplete follow up of patients at the end of therapy (after 12 months of the combined therapy in genotype 4 which is the most common type in our country) (24, 25), and shortage of better investigation methods for the detection of systolic and diastolic dysfunction (e.g., tissue Doppler, radionuclide gated blood pool). Thus, it is advisable that future studies use these methods in the diagnosis of a larger number of patients who complete the full course of interferon therapy. The results of our study suggest that combination therapy for the treatment of chronic hepatitis C is not toxic to the cardiovascular system and it may be used safely in patients who do not have pre-existing cardiac disease.
